# Supplementary material for: Sedimentary DNA insights into Holocene Adélie penguin (Pygoscelis adeliae) populations and ecology in the Ross Sea, Antarctica
Source: Nat Commun. 2025 Mar 5;16:1798. doi: 10.1038/s41467-025-56925-4 (PMC11883008; doi:10.1038/s41467-025-56925-4)
Supplement: Supplementary file 1 — Supplementary Information [file 41467_2025_56925_MOESM1_ESM.pdf]

Supplementary Tables and Figures

Sedimentary DNA insights into Holocene Adélie penguin (*Pygoscelis adeliae*) populations and ecology in the Ross Sea, Antarctica

Jamie R. Wood[1,2*], Chengran Zhou[3,4*], Theresa L. Cole[5], Morgan Coleman[5], Dean P. Anderson[5], Phil O'B. Lyver[5], Shangjin Tan[6,3], Xueyan Xiang[3,4], Xinrui Long[3,7], Senyu Luo[6,3], Miao Lou[8], John R. Southon[9], Qiye Li[3,4], Guojie Zhang[10,11,12]

[1]Australian Centre for Ancient DNA, School of Biological Sciences, North Terrace Campus, University of Adelaide, South Australia 5005, Australia; [2]Environment Institute, University of Adelaide, North Terrace Campus, South Australia 5005, Australia; [3]BGI Research, Wuhan 430074,China; [4]State Key Laboratory of Agricultural Genomics, BGI Research, Shenzhen 518083, China; [5]Manaaki Whenua Landcare Research, PO Box 69040, Lincoln 7640, New Zealand; [6]College of Life Sciences, University of Chinese Academy of Sciences, Beijing 100049, China; [7]Guangdong Provincial Key Laboratory of Marine Biotechnology, Institute of Marine Sciences, Shantou University, Shantou 515063, China; [8]College of Life Sciences, Wuhan University, Wuhan 430072, China ; [9]Department of Earth System Science, University of California-Irvine, Irvine, California 92697, USA; [10]Center for Evolutionary & Organismal Biology and Women's Hospital at Zhejiang University School of Medicine, and Liangzhu Laboratory, Zhejiang University Medical Center, Hangzhou, China. [11]Liangzhu Laboratory, Zhejiang University Medical Center, Hangzhou, China. [12]Villum Center for Biodiversity Genomics, Section for Ecology and Evolution, Department of Biology, University of Copenhagen, Copenhagen 2100, Denmark.

Supplementary Tables

Supplementary Table 1. Details of pits excavated at Adélie penguin colonies on Ross Island and Victoria Land Coasts, western Ross Sea, Antarctica, 2019-2020.

Region	Site	Pit	Latitude	Longitude	Excavation dates	Area (m^2)	Depth (mm)	Status	Age class	Colony size (estimated breeding pairs)
Northern	Cape Adare	2	71.30219444 S	170.22363889 E	13 January 2019	0.09	230	Active	Active	338,231[1]
Northern	Cape Adare	1	71.30258333 S	170.22005556 E	13 January 2019	0.09	230	Active	Active	338,231[1]
Northern	Cape Hallett	1	72.32191667 S	170.22511111 E	10-11 January 2019	0.50	600	Active	Active	19,740[2]
Mid	Campo Icaro	1	74.71319444 S	164.11202778 E	5 January 2019	0.75	530	Abandoned	Mid Holocene	-
Mid	North Adélie Cove	1	74.73469444 S	164.01433333 E	6 January 2019	0.50	400	Abandoned	Mid Holocene	-
Mid	Adélie Cove	1	74.76769444 S	164.10150000 E	8 January 2019	0.09	240	Active	Active	11,438[3]
Southern	Cape Bird	1	77.2148166 S	166.433833 E	25-26 January 2020	1.00	770	Active	Active	59,192[2]
Southern	Marble Point	2	77.4302333 S	163.833717 E	23 January 2020	0.375	150	Abandoned	Late Holocene	-
Southern	Marble Point	1	77.43085 S	163.826917 E	22 January 2020	0.60	180	Abandoned	Late Holocene	-
Southern	Cape Crozier	1	77.4537833 S	169.21525 E	3 February 2020	0.50	370	Active	Active	249,386[2]
Southern	Cape Royds	1	77.5529333 S	166.160067 E	20 January 2020	0.25	250	Active	Active	2,667[2]
Southern	Cape Royds	2	77.5537 S	166.162117 E	21 January 2020	0.30	520	Active	Active	2,667[2]
Southern	Cape Barne	1	77.5758 S	166.242 E	30 January 2020	0.50	190	Abandoned	Recently abandoned	-
Southern	Cape Barne	2	77.5777833 S	166.2409 E	1 February 2020	0.09	150	Abandoned	Recently abandoned	-

Supplementary Table 2. Radiocarbon dates for Adélie penguin eggshell sampled from pits on Ross Island and Victoria Land Coasts, western Ross Sea, Antarctica, 2019-2020.

UCIAMS #	Locality	Depth	^{14}C age	1-sigma	Median cal. age BP	95.4% range
229710	Marble Point pit 1	Spit 1: 0-2cm	3820	20	2715	3001-2385
229711	Marble Point pit 1	Spit 4: 12-18cm	3895	15	2811	3107-2494
229712	Marble Point pit 2	Spit 6: 10-13cm	4625	35	3922	4152-3686
229713	Cape Royds pit 1	Spit 3: 3-7cm	1255	25	83	257-modern
229716	Cape Royds pit 2	Spit 4: 9-12cm	1060	20	56	211-modern
229703	Cape Barne pit 1	Spit 1: 0-2cm	1400	35	126	353-modern
229701	Cape Barne pit 1	Spit 6: 15-19cm	1470	15	156	384-modern
229704	Cape Barne pit 2	Spit 2: 0-3cm	1415	15	129	359-modern
229702	Cape Barne pit 2	Spit 5: 9-15cm	1410	15	127	345-modern
229699	Cape Crozier	Spit 9: 34-37cm	1190	20	70	242-modern
229709	Cape Bird	Spit 7: 15-18cm	1350	15	104	297-modern
229698	Adelie Cove	Spit 6: 19-24cm	835	20	41	135-modern
229715	North Adelie Cove	Spit 2: 5-10cm	5930	60	5251	5544-4947
229717	North Adelie Cove	Spit 6: 20-25cm	6180	60	5575	5921-5236
229695	Campo Icaro	Spit 1: 0-5 cm	4200	60	3095	3422-2749
229714	Campo Icaro	Spit 3: 10-15 cm	6260	50	5662	5978-5317
229696	Cape Hallett	Spit 1: 3-7cm	1375	15	114	323-modern
229697	Cape Hallett	Spit 10: 47-52cm	1450	20	146	376-modern
229705	Cape Adare pit 1	Spit 7: 17-20cm	925	15	44	152-modern
229700	Cape Adare pit 2	Spit 8: 23-25cm	1555	15	225	444-modern

Supplementary Table 3. Number of reads assigned to Eukaryota kingdoms/groups by the mitochondrial mapping and ngsLCA methods for taxonomic assignment.

Site	Method	Fungi	Metazoa	Viridiplantae	Protists
Cape Adare2	mito	94	814	776	504
	ngsLCA	4093	190808	5363	3239
Cape Adare 1	mito	2236	1534	8227	5126
	ngsLCA	44690	350229	43006	39392
Cape Hallett	mito	60	444	1054	606
	ngsLCA	25667	72871	21726	18877
Campo Icaro	mito	70	201	543	285
	ngsLCA	7640	38822	4712	2612
North Adélie Cove	mito	175	201	1225	491
	ngsLCA	10643	48999	9411	5693
Adélie Cove	mito	180	275	617	322
	ngsLCA	13049	71979	18016	10051
Cape Bird	mito	2267	1331	1302	752
	ngsLCA	32141	469184	27236	12184
Cape Crozier	mito	79	2740	872	612
	ngsLCA	6105	1487571	7326	3966
Marble Point 1	mito	22	830	2284	2423
	ngsLCA	10751	144441	10729	1476
Marble Point 2	mito	17	292	2270	2565
	ngsLCA	1389	52350	5405	1147
Cape Royds 1	mito	26	1270	2959	1344
	ngsLCA	4595	188416	7498	5349
Cape Royds 2	mito	89	1383	3455	2630
	ngsLCA	41457	285086	70912	125494
Cape Barne 1	mito	257	1555	536	351
	ngsLCA	767	86979	1211	193
Cape Barne 2	mito	60	1732	2686	3061
	ngsLCA	1089	316263	4418	1032

Supplementary Table 4. Number of reads assigned to major Eukaryote phyla identified by the mitochondrial mapping and ngsLCA methods for taxonomic assignment.

Kingdom/group	Phylum	mito	ngsLCA
Fungi	Ascomycota	5287	127692
	Microsporidia	216	1149
	Basidiomycota	108	41158
	Mucoromycota	0	34128
	Other	47	2077
Metazoa	Chordata	14314	3625310
	Other	325	218072
Viridiplantae	Chlorophyta	7581	21364
	Prasinodermophyta	22	0
	Streptophyta	21230	218072
Protists	Ciliophora	89	65211
	Oomycota	140	64470
	Euglenozoa	80	46008
	Apicomplexa	54	14604
	Evosea	92	11165
	Preaxostyla	0	7415
	Rhodophyta	2305	5771
	Bacillariphyta	6897	5344
	Heterolobosea	11067	3778
	Perkinsozoa	0	2362
	Fornicata	0	805
	Cercozoa	118	459
	Discosea	88	258
	Endomyxa	12	100
	Parabasalia	0	60
	Foraminifera	0	20
	Haptista	12	0
	Imbricatea	6	0
	Tubulinea	113	0

Supplementary Table 5. Summed totals of Adélie penguin mitochondrial control regions reads recovered from each excavation pit and lineage assignments (RS = Ross Sea, A=Antarctic).

Pit	n RS lineage	n A lineage	n unassigned to lineage	% RS lineage of assigned reads	% A lineage of assigned reads
Cape Barne 2	1	28	14	3.45	96.55
Cape Barne 1	0	9	5	0	100.00
Cape Royds 2	1	23	29	4.17	95.83
Cape Royds 1	0	21	23	0	100.00
Cape Crozier	20	197	118	9.22	90.78
Marble Point 2	0	2	0	0	100.00
Marble Point 1	3	10	10	23.08	76.92
Cape Bird	4	49	35	7.55	92.45
Adélie Cove	1	4	2	20.00	80.00
North of Adélie Cove	0	7	0	0	100.00
Camp Icaro	0	1	5	0	100.00
Cape Hallett	2	3	2	40.00	60.00
Cape Adare 2	2	10	7	16.67	83.33
Cape Adare 1	2	23	16	8.00	92.00

Supplementary Table 6. Taxa identified in sedaDNA from Ross Island and Victoria Land Coasts, western Ross Sea, Antarctica, that were considered to represent marine plankton.

Phylym	Class	Family	Notes
Bacillariophyta			Diatoms. A range of marine taxa were identified, with reads being dominated by the marine genus *Berkeleya*.
Cercozoa			All reads were assigned to order Chlorarachniophyceae, which are exclusively marine.
Chlorophyta	Chlorodendrophyaceae		Aquatic, including planktonic taxa.
Chlorophyta	Chlorophyaceae		Important constituents of marine phytoplankton.
Chlorophyta	Chloropicophyceae		Marine plankton taxa.
Chlorophyta	Mamiellophyceae		Key phytoplankton in coastal waters.
Chlorophyta	Nephroselmidophyceae		Nephroselmis are all marine except one.
Chlorophyta	Picocystophyceae		Saline.
Chlorophyta	Pyramimonadophyceae		Marine.
* Chlorophyta - all classes were included except Trebouxiophyaceae (mostly terrestrial and freshwater) and Ulvophyaceae (benthic macroalgae)			
Ciliophora	Oligohymenophorea		Marine, including the identified family Uronematidae.
Ciliophora	Spirotrichea		Spirotrichea commonly marine. Identified families Halteriidae and Strombidiidae are coastal and marine planktonic.
Haptista			Coccoliths. Identified genus *Emiliania* in most oceans.
Heterolobosea		Tulamoebidae	Halophyte, marine.
Heterolobosea		Gruberellidae	Marine or soil.
Heterolobosea		Vahlkampfiidae	Widespread, including marine and saline.
* Heterolobosea – the phylum Discosea was omitted as it can be marine, but the identified taxa within it were ambiguous. The family Acrasidae was excluded due to terrestrial habitat.			
Microsporidia			Not strictly plankton, but included as parasites whose spores spread by currents.
phylum NA	Bigyra		Marine protists.
phylum NA	Bolidophyceae		Photosynthetic heterokont picophytoplankton.
phylum NA	Chrysophyceae		Identified genus *Ochromonas* is littoral halotolerant.
phylum NA	Cryptophyceae		Most identified families are aquatic and coastal.
phylum NA	Eustigmatophyceae		Identified genera are marine.
phylum NA	Raphidophyceae		All reads identified as Chattonellaceae, a marine algae.
phylum NA	Synurophyceae		Silicious scaled algae.
Prasinodermophyta			Identified genus found in ocean.
Rhodophyta	Bangiophyceae		Single cell red algae, marine.

Supplementary Figures

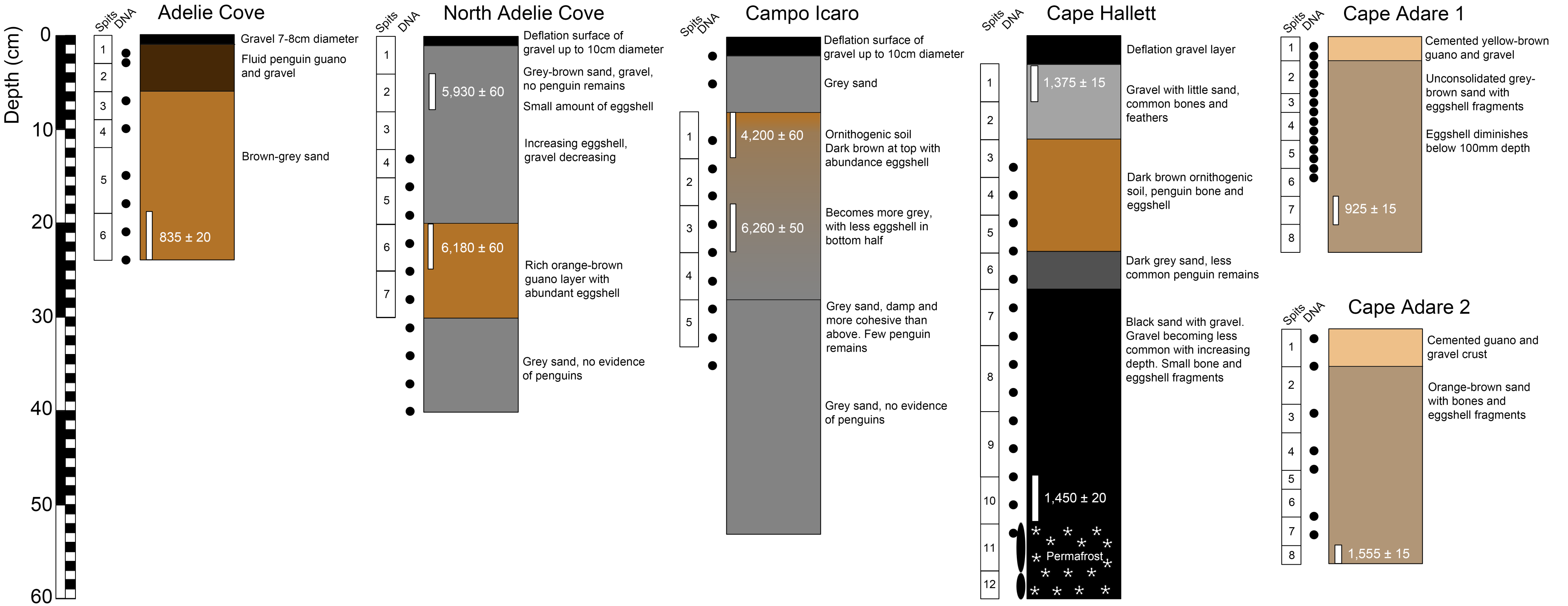

Supplementary Figure 1 | Stratigraphic details of pits from the mid- to northern Ross Sea. Excavations at Adélie penguin colonies located on the mid- to northern Victoria Land Coast, western Ross Sea, Antarctica, indicating bulk sediment spits collected, location at which samples for *seda*DNA analyses were taken and radiocarbon dates.

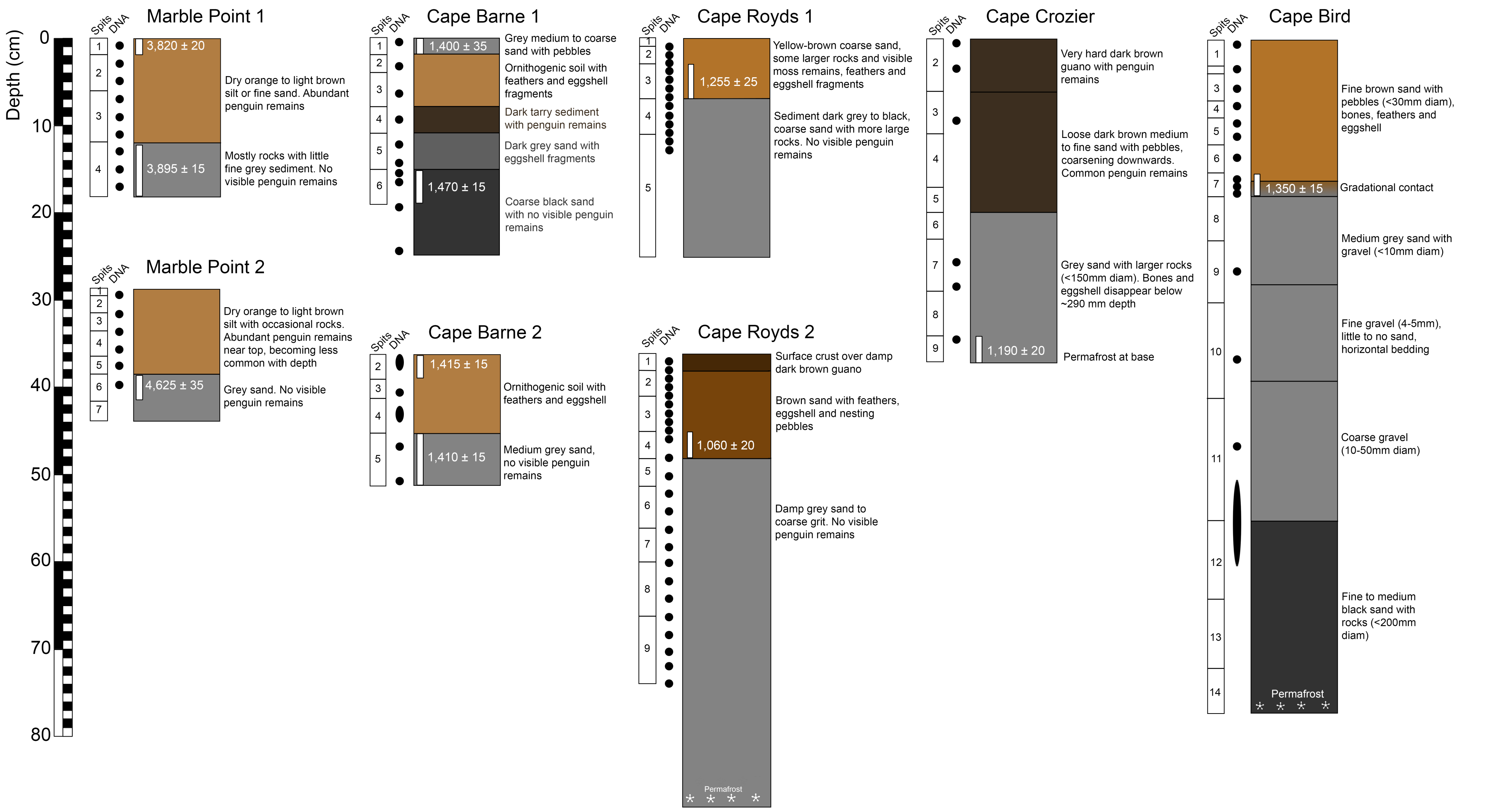

Supplementary Figure 2 | Stratigraphic details of pits from the southern Ross Sea. Excavations at Adélie penguin colonies located on the Ross Island and southern Victoria Land Coasts, western Ross Sea, Antarctica, indicating bulk sediment spits collected, location at which samples for *seda*DNA analyses were taken and radiocrbon dates.

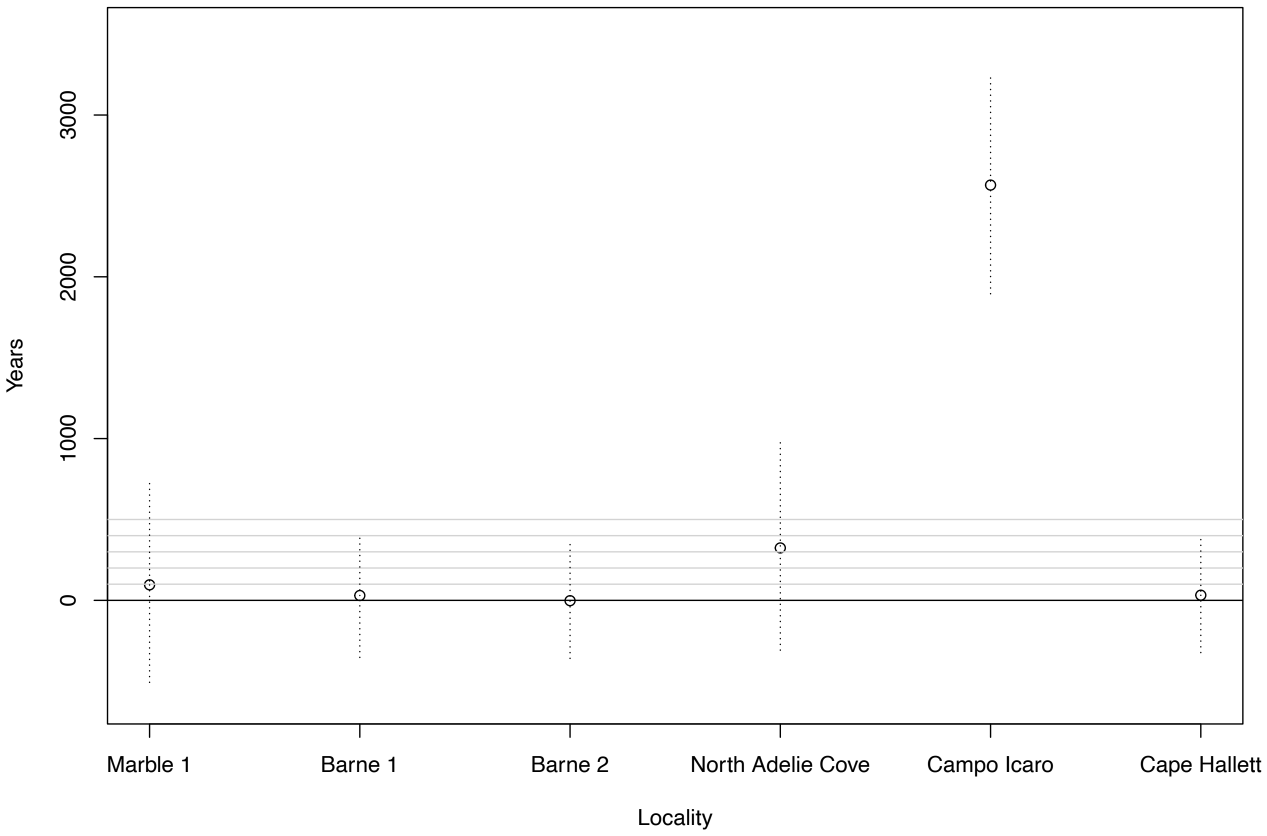

Supplementary Figure 3 | Offset between calibrated radiocarbon dates. Offset between calibrated radiocarbon dates for pits where two dates were obtained, as a proxy for sediment accumulation time. Circles represent differences between median ages, while the vertical dashed lines represent the range of possible offset represented by the 95% confidence intervals.

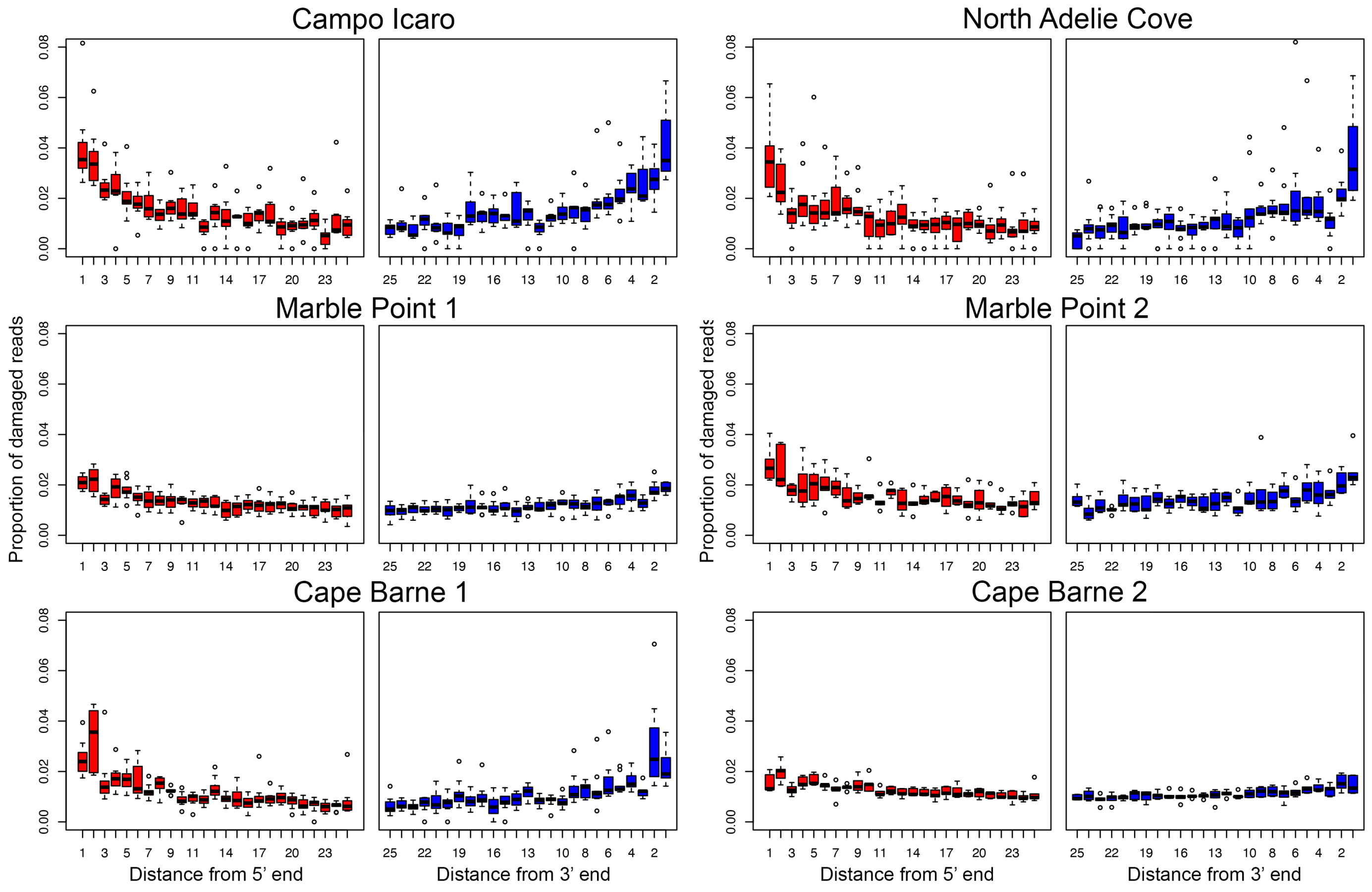

Supplementary Figure 4. **Deamination profiles for Adélie penguin DNA from abandoned colonies.** Deamination profiles for Adélie penguin (*Pygoscelis adeliae*) genomic DNA retrieved from sediment samples from pits excavated at abandoned Adélie penguin colonies, eastern Victoria Land and Ross Island, Antarctica. Boxplots show the distribution of the proportion of deaminated reads at each position from the end of DNA fragments pooled across all sediment samples in each pit.

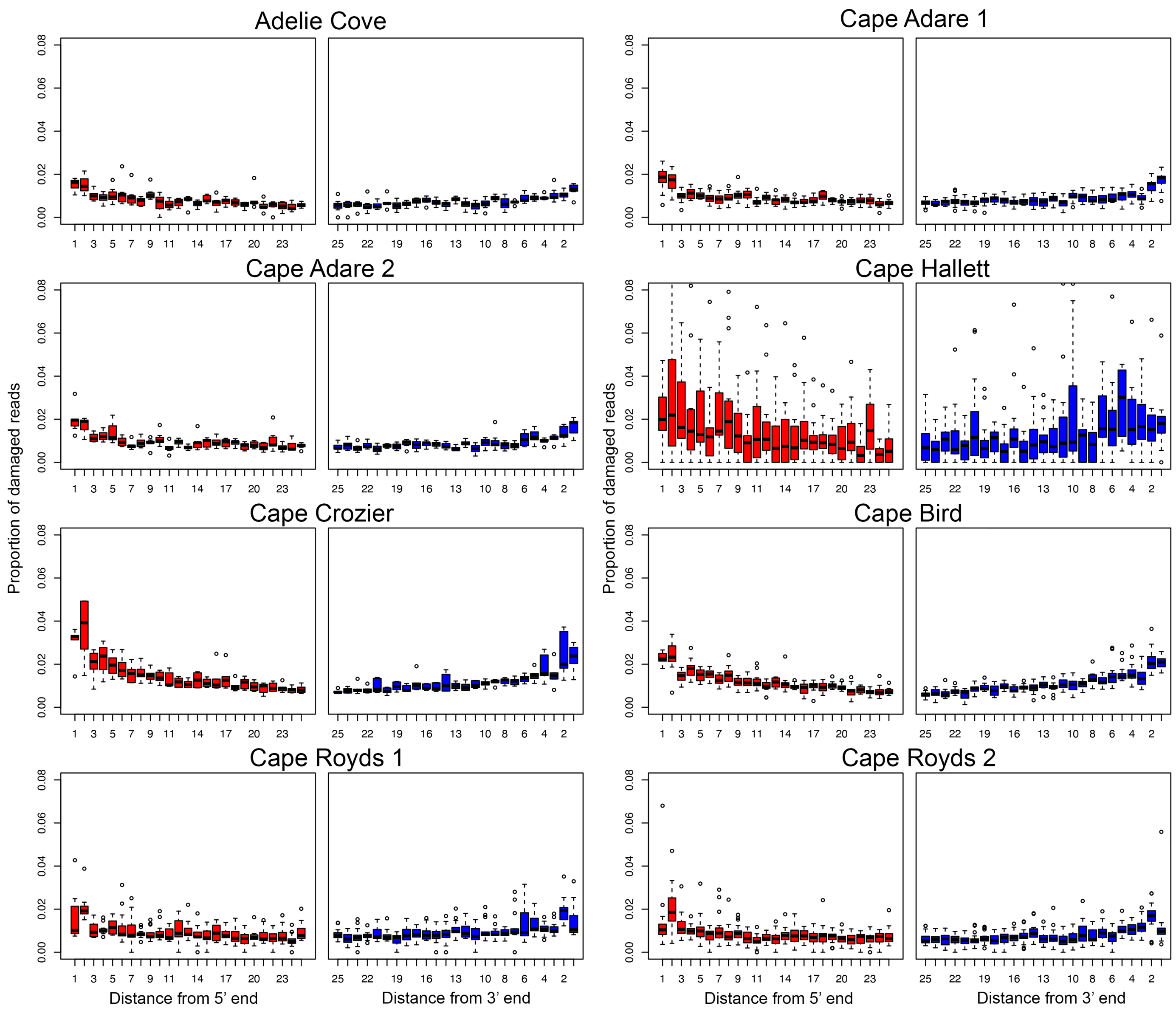

Supplementary Figure 5. **Deamination profiles for Adélie penguin DNA from active colonies.** Deamination profiles for Adélie penguin (*Pygoscelis adeliae*) genomic DNA retrieved from sediment samples from pits excavated at active Adélie penguin colonies, eastern Victoria Land and Ross Island, Antarctica. Boxplots show the distribution of the proportion of deaminated reads at each position from the end of DNA fragments pooled across all sediment samples in each pit.

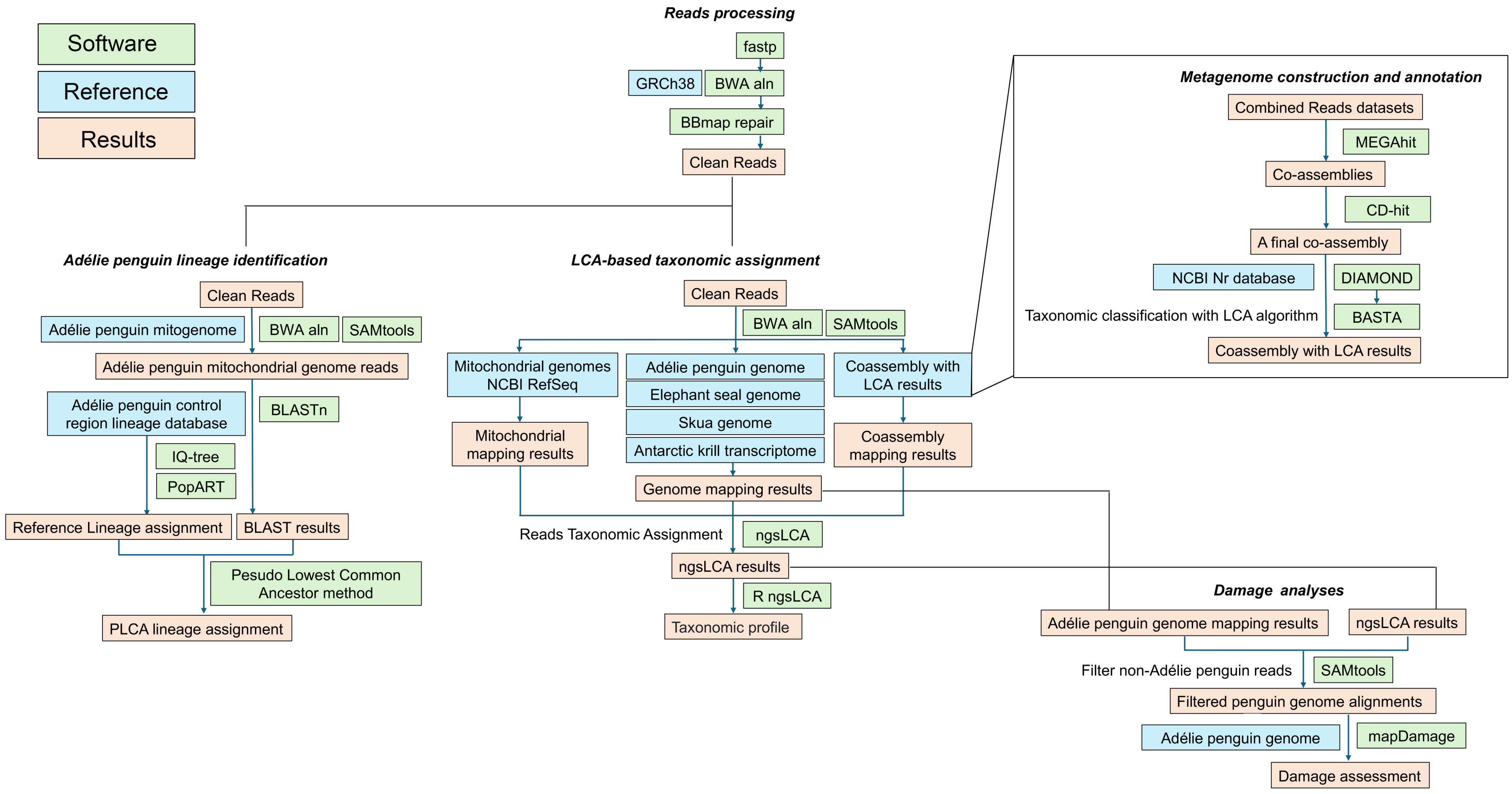

Supplementary Figure 6. **Bioinformatic workflow for analysis of sedaDNA metagenomes.** Bioinformatic workflow for analysis of sedaDNA metagenomes from Ross Island and Victoria Land Coasts, western Ross Sea, Antarctica**.**

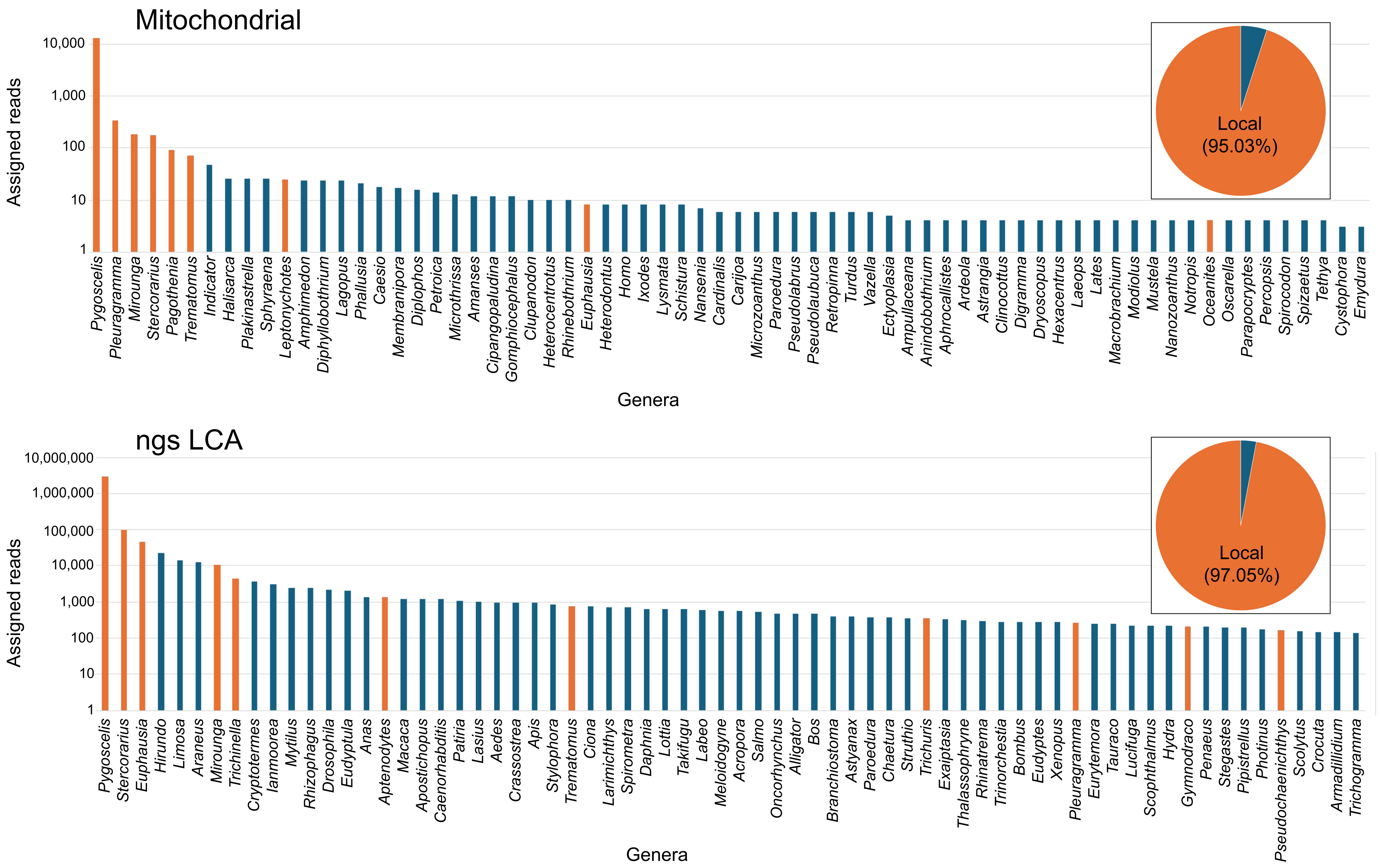

Supplementary Figure 7 | Geographic range of identified taxa. Representation of local (to Ross Sea region of Antarctica) vs non-local genera among those identified by the two taxonomic assignment methods.

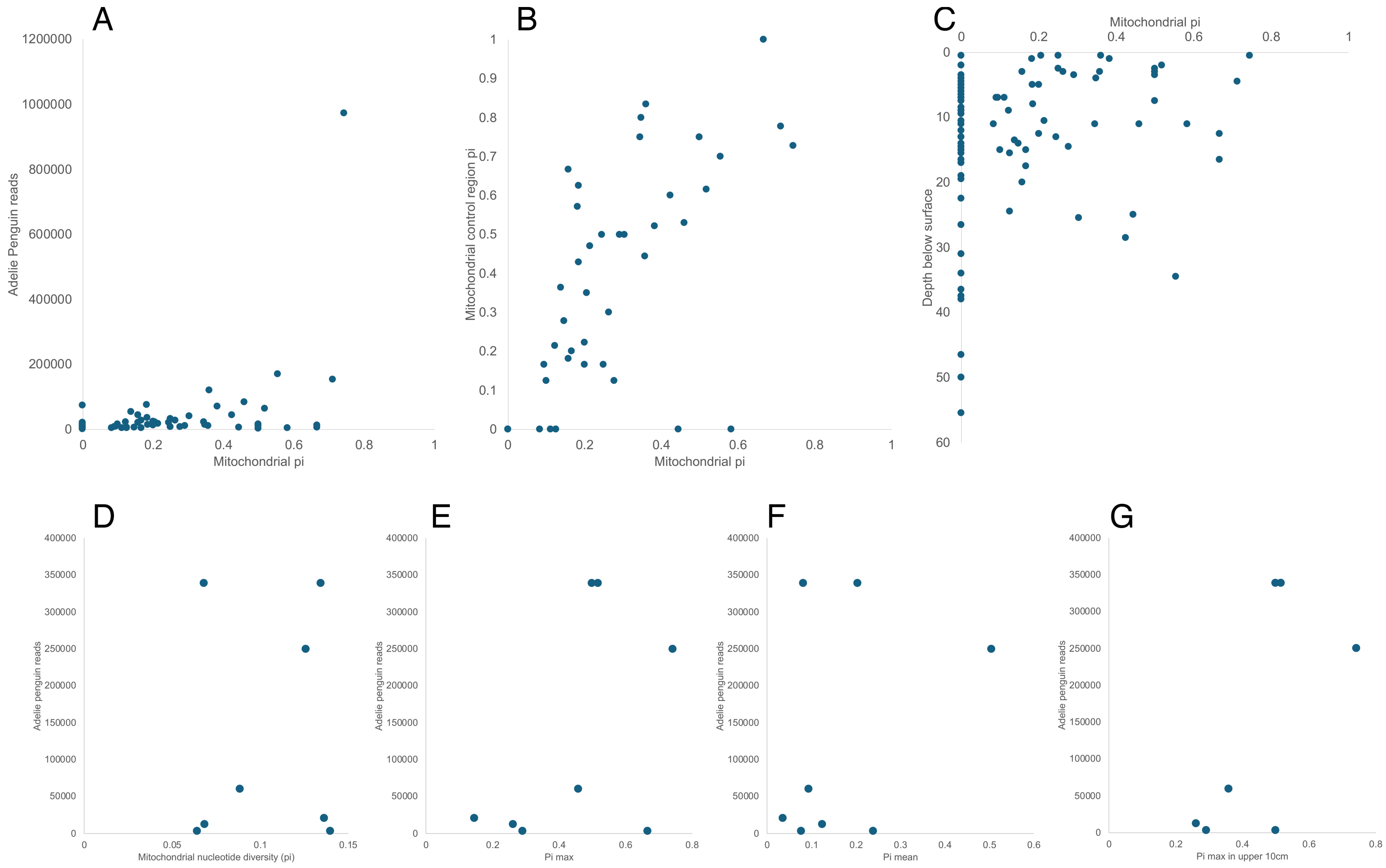

Supplementary Figure 8. Adélie penguin mitochondrial nucleotide diversity. Relationships between mitochondrial nucleotide diversity (pi) of each sample and A) Number of mapped Adélie penguin reads; B) Mitochondrial control region nucleotide diversity; C) Depth below surface. Relationships between number of mapped Adélie penguin reads from each site and D) Mitochondrial nucleotide diversity (pi); E) Maximum observed pi; F) Mean pi; G) Maximum observed pi in uppermost 10cm of stratigraphy.

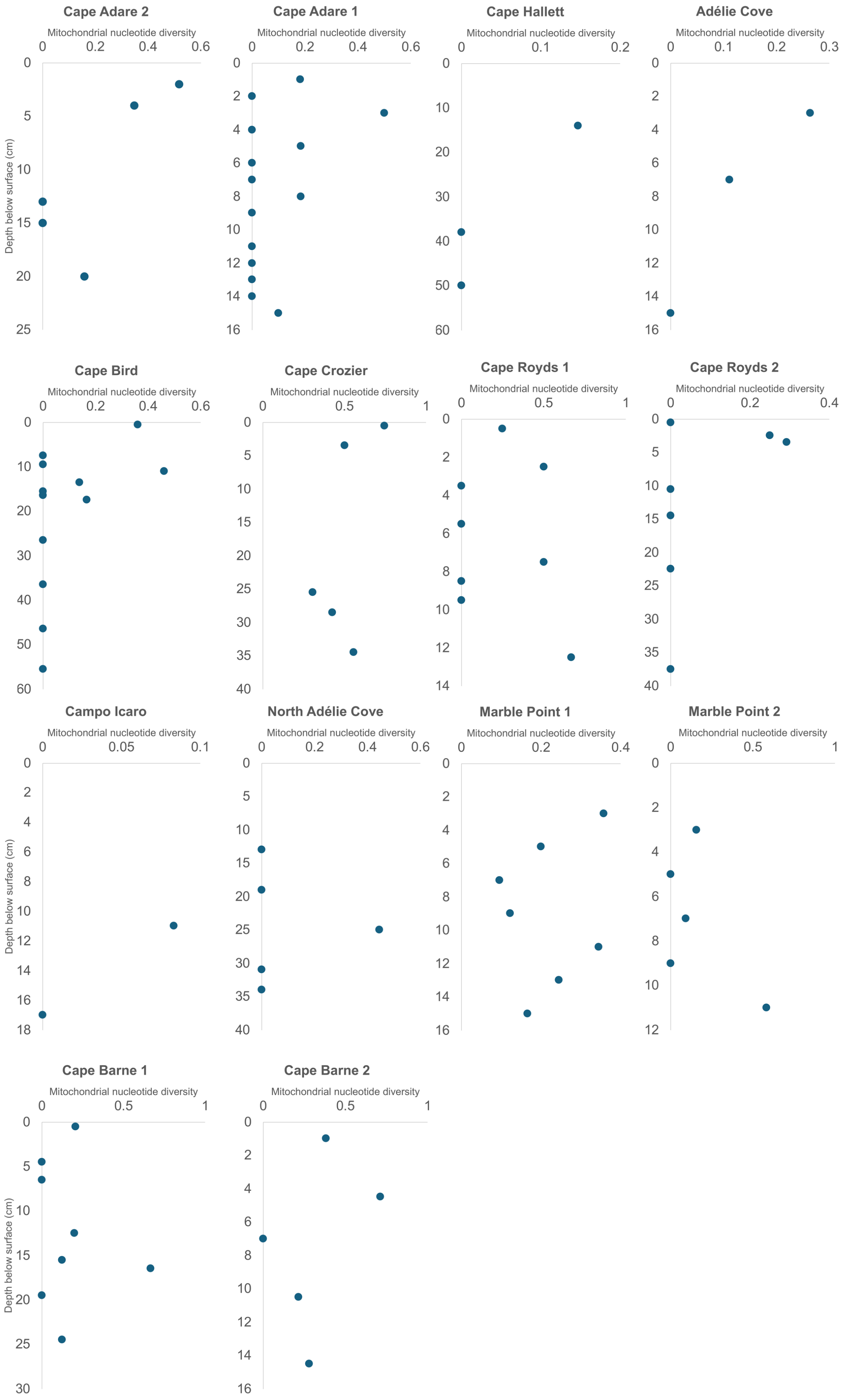

Supplementary Figure 9 | Adélie penguin mitochondrial nucleotide diversity vs sample depth. Relationships between mitochondrial nucleotide diversity and sediment sample depth below surface. Top row are active colonies from the mid-northern Ross Sea, second row are active colonies from the southern Ross Sea. Bottom two rows are abandoned colonies.

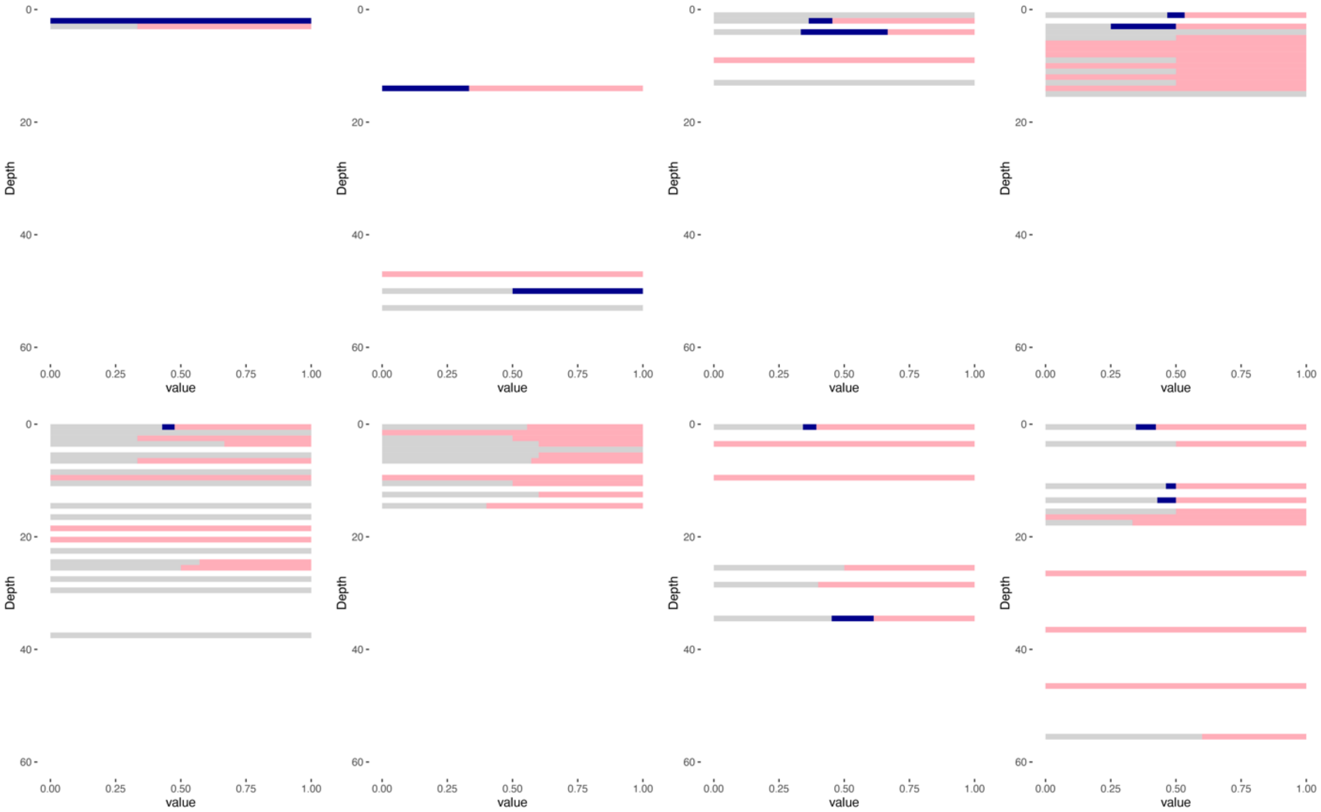

Supplementary Figure 10. Stratigraphic proportions of mitochondrial control region reads from *seda*DNA at active Adélie penguin colonies. Stratigraphic proportions of reads from sedaDNA at active Adélie penguin (*Pygoscelis adeliae*) colonies that mapped to Adélie penguin mitochondrial control region and were assigned to Antarctic lineage (pink) and Ross Sea lineage (blue). Unassigned reads are shown in grey. Top row are mid-northern Ross Sea colonies (left to right: Adélie Cove, Cape Hallett, Cape Adare 2, Cape Adare 1). Bottom row are southern Ross Sea colonies (left to right: Cape Royds 2, Cape Royds 1, Cape Crozier, Cape Bird).

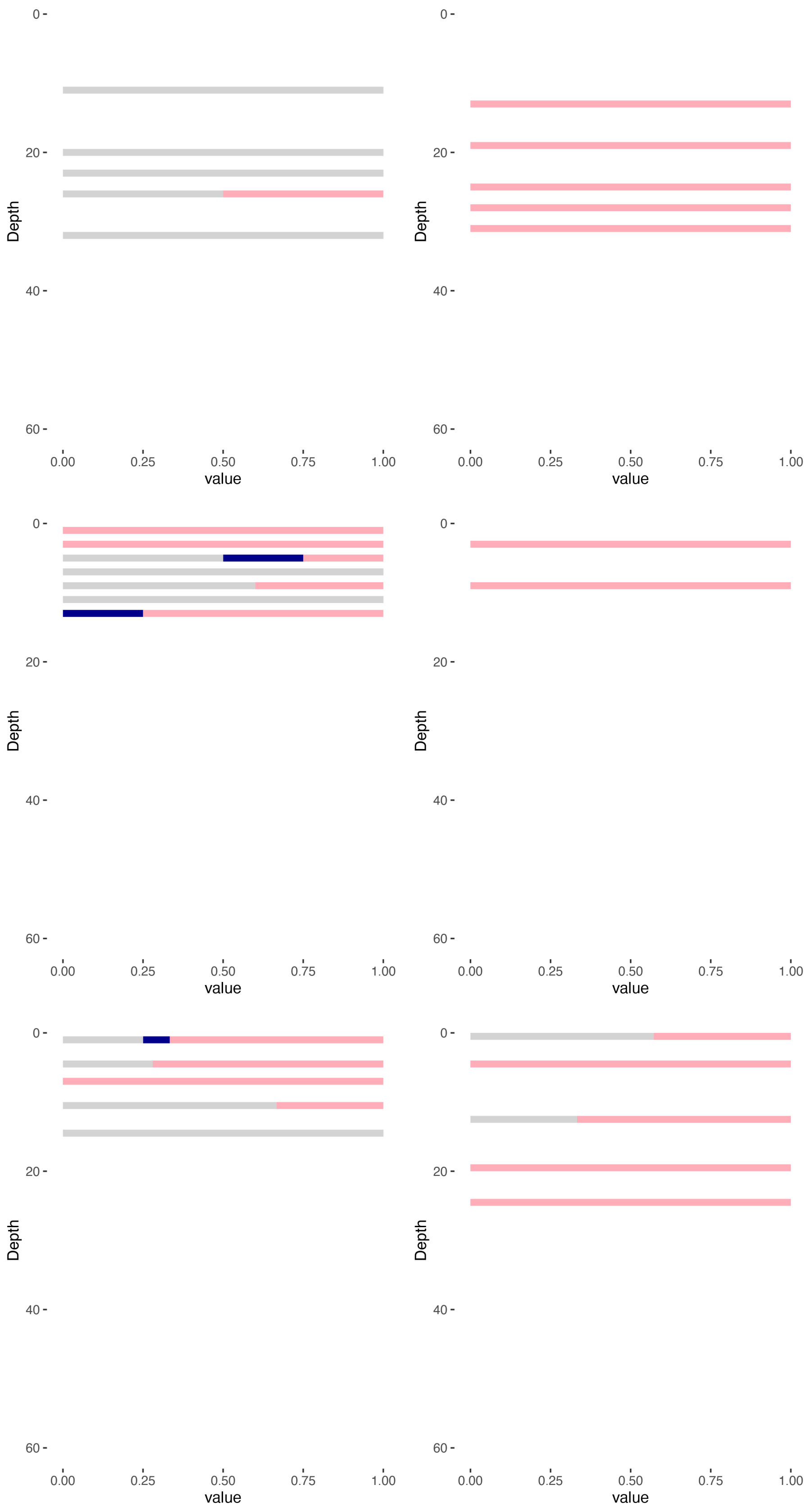

Supplementary Figure 11. Stratigraphic proportions of mitochondrial control region reads from *seda*DNA at abandoned Adélie penguin colonies. Stratigraphic proportions of reads from sedaDNA at abandoned Adélie penguin (*Pygoscelis adeliae*) colonies that mapped to Adélie penguin mitochondrial control region and were assigned to Antarctic lineage (pink) and Ross Sea lineage (blue). Unassigned reads are shown in grey. Top row are mid-Holocene colonies (left to right: Campo Icaro, North Adélie Cove); Middle Row are late Holocene colonies (left to right: Marble Point 1, Marble Point 2); Bottom Row are recently abandoned colonies (left to right: Cape Barne 2, Cape Barne 1.

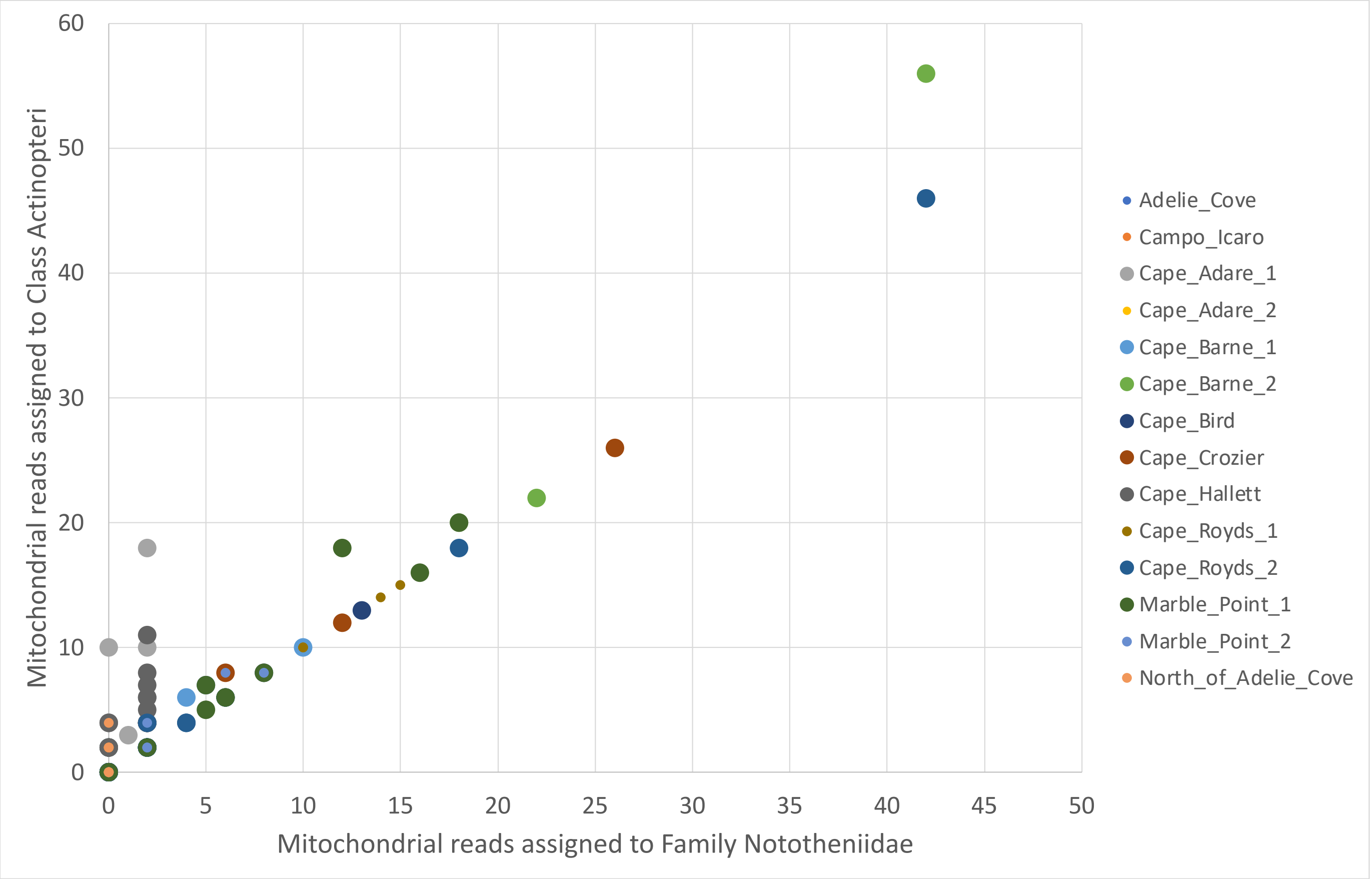

Supplementary Figure 12 | Representation of Antarctic fish (Nototheniidae) among fish mitochondrial reads. Correlation between the number of reads mapped to fish (Actinopteri) mitogenomes in each sample and the number of these that were resolved to the Antarctic fish family Nototheniidae.

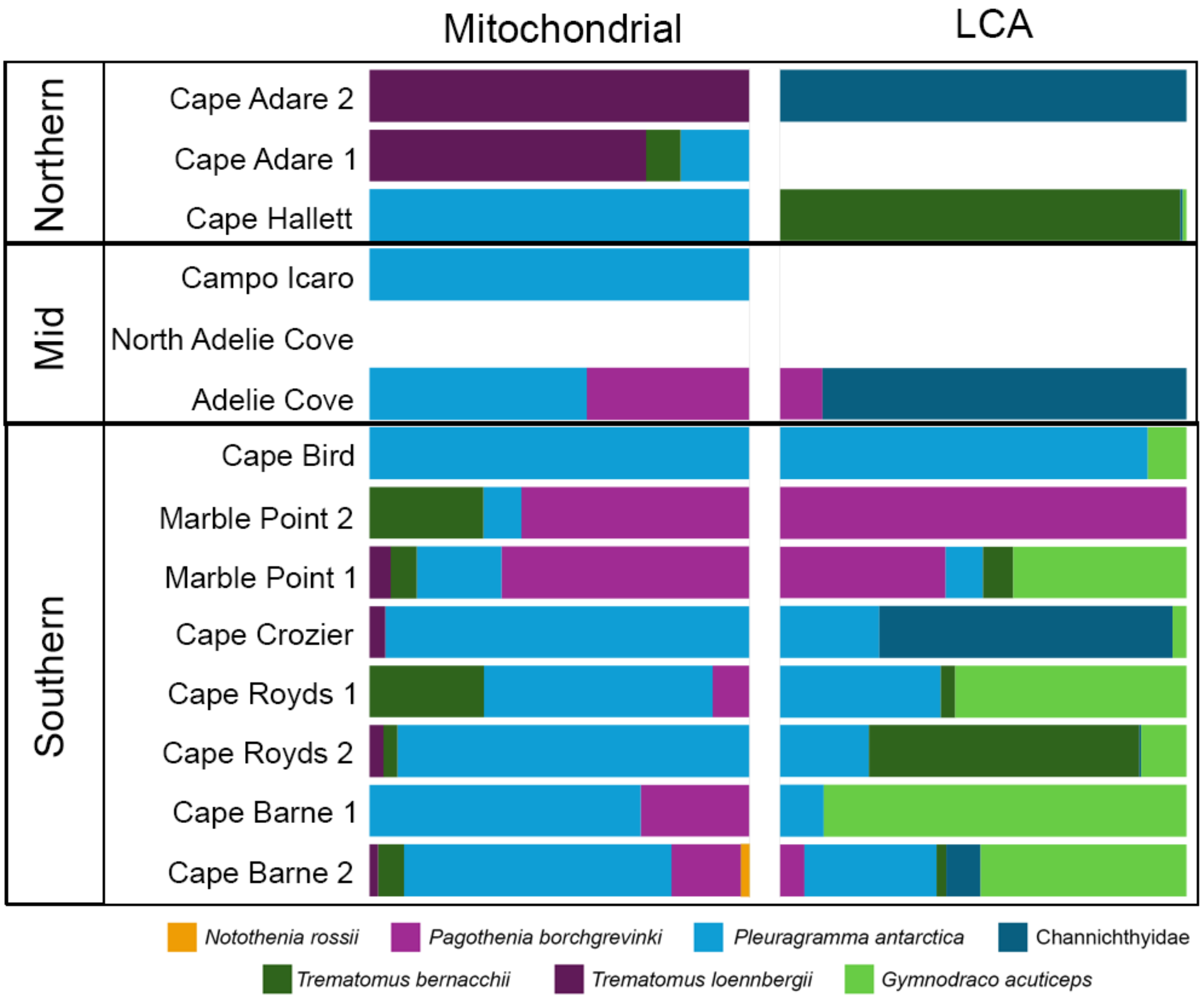

Supplementary Figure 13 | Relative proportions of Antarctic fish species. Relative proportions of Antarctic fish species detected using mitochondrial and LCA approaches to taxonomic assignment. Sites are arranged from north to south, and grouped within different latitudinal regions of the Ross Sea.

Supplementary References

[1] Lynch, H.J., LaRue, M.A. First global census of the Adélie penguin. *Auk. Ornitholog. Adv*. **131**, 457–466 (2014).

[2] LaRue, M.A., *et al*. A method for estimating colony sizes of Adélie penguins using remote sensing imagery. *Polar Biology* **37**, 507-517 (2014).

[3] Olmastroni, S., Feretti, F., Burrini, L., Ademollo, N., Fattorini, N. Breeding ecology of Adélie penguins in Mid Victoria Land, Ross Sea Antarctica. *Diversity* **14**, 429 (2022).
