# Supplementary material for: Sedimentary DNA insights into Holocene Adélie penguin (Pygoscelis adeliae) populations and ecology in the Ross Sea, Antarctica
Source: Nat Commun. 2025 Mar 5;16:1798. doi: 10.1038/s41467-025-56925-4 (PMC11883008; doi:10.1038/s41467-025-56925-4)
Supplement: Supplementary file 3 — Description of Additional Supplementary Files [file 41467_2025_56925_MOESM3_ESM.pdf]

Description of Additional Supplementary Files

Supplementary Code 1. Code used in sequence data processing, metagenome construction and annotation, mitogenome mapping and annotation, mitochondrial SNP calling and pi calculation, Adélie penguin lineage identification and LCA-based taxonomic assignment.

Supplementary Data 1. Key metadata for individual sample sequencing libraries, with high level results from the LCA taxonomic assignment of reads. Group refers to the subsets of samples which were grouped together for co-assembly.

Supplementary Data 2. Adélie penguin (*Pygoscelis adeliae*) genome mapped read length counts.

Supplementary Data 3. Adélie penguin (*Pygoscelis adeliae*) mitochondrial nucleotide diversity (pi) obtained from sediment samples from colonies within on Ross Island and Victoria Land Coasts, western Ross Sea, Antarctica.

Supplementary Data 4. Read abundance for taxa considered to represent marine plankton in sedaDNA from Ross Island and Victoria Land Coasts, western Ross Sea, Antarctica.

Supplementary Data 5. Sequences for adapters and primers use in library preparation.
