# Supplementary material for: Sedimentary DNA insights into Holocene Adélie penguin (Pygoscelis adeliae) populations and ecology in the Ross Sea, Antarctica
Source: Nat Commun. 2025 Mar 5;16:1798. doi: 10.1038/s41467-025-56925-4 (PMC11883008; doi:10.1038/s41467-025-56925-4)
Supplement: Supplementary file 6 — Reporting Summary [file 41467_2025_56925_MOESM6_ESM.pdf]

nature portfolio

Corresponding author(s): Jamie R Wood
Guojie Zhang

Last updated by author(s): 10 Jan 2025

Reporting Summary

Nature Portfolio wishes to improve the reproducibility of the work that we publish. This form provides structure for consistency and transparency in reporting. For further information on Nature Portfolio policies, see our Editorial Policies and the Editorial Policy Checklist.

Statistics

For all statistical analyses, confirm that the following items are present in the figure legend, table legend, main text, or Methods section.

n/a	Confirmed	
☐	☒	The exact sample size (n) for each experimental group/condition, given as a discrete number and unit of measurement
☒	☐	A statement on whether measurements were taken from distinct samples or whether the same sample was measured repeatedly
☐	☒	The statistical test(s) used AND whether they are one- or two-sided<br>*Only common tests should be described solely by name; describe more complex techniques in the Methods section.*
☒	☐	A description of all covariates tested
☒	☐	A description of any assumptions or corrections, such as tests of normality and adjustment for multiple comparisons
☐	☒	A full description of the statistical parameters including central tendency (e.g. means) or other basic estimates (e.g. regression coefficient) AND variation (e.g. standard deviation) or associated estimates of uncertainty (e.g. confidence intervals)
☐	☒	For null hypothesis testing, the test statistic (e.g. F, t, r) with confidence intervals, effect sizes, degrees of freedom and P value noted<br>*Give P values as exact values whenever suitable.*
☒	☐	For Bayesian analysis, information on the choice of priors and Markov chain Monte Carlo settings
☒	☐	For hierarchical and complex designs, identification of the appropriate level for tests and full reporting of outcomes
☒	☐	Estimates of effect sizes (e.g. Cohen's d, Pearson's r), indicating how they were calculated

Our web collection on statistics for biologists contains articles on many of the points above.

Software and code

Policy information about availability of computer code

Data collection	The following programs and online tools were used: OxCal Online 4.4, fastp v.0.22.0, BWA v0.7.17-r1188, bbMAP v.20150602, trf v.3, MEGAhit v.1.2.9, DIAMOND v.2.0.9.147, BASTA v.1.4, BLASTn v.2.12.0, Picard v.2.25.1, SAMtools v.1.17, BamDeal v.0.26, ngsLCA v.1.0.0, mapDamage v.2.1.0, MAFFT v.7.487, PopART v.3, Makeblastdb v.2.11.0
Data analysis	Published tools were used as described in the methods section, and scripts can be found in the Supplementary Code.

For manuscripts utilizing custom algorithms or software that are central to the research but not yet described in published literature, software must be made available to editors and reviewers. We strongly encourage code deposition in a community repository (e.g. GitHub). See the Nature Portfolio guidelines for submitting code & software for further information.

Data

Policy information about availability of data

All manuscripts must include a data availability statement. This statement should provide the following information, where applicable:

- Accession codes, unique identifiers, or web links for publicly available datasets
- A description of any restrictions on data availability
- For clinical datasets or third party data, please ensure that the statement adheres to our policy

The source sequencing data generated in this study, with tag look-up information, have been deposited in the CNSA of the CNGBdb database under the accession

number CNP0002256 [https://db.cngb.org/search/project/CNP0002256/] and in the NCBI as BioProject PRJNA1202586 [https://www.ncbi.nlm.nih.gov/bioproject/1202586]. The locations and depths of samples from which these data were generated are provided in Supplementary Data 1.

Research involving human participants, their data, or biological material

Policy information about studies with human participants or human data. See also policy information about sex, gender (identity/presentation), and sexual orientation and race, ethnicity and racism.

Reporting on sex and gender	*Use the terms sex (biological attribute) and gender (shaped by social and cultural circumstances) carefully in order to avoid confusing both terms. Indicate if findings apply to only one sex or gender; describe whether sex and gender were considered in study design; whether sex and/or gender was determined based on self-reporting or assigned and methods used. Provide in the source data disaggregated sex and gender data, where this information has been collected, and if consent has been obtained for sharing of individual-level data; provide overall numbers in this Reporting Summary. Please state if this information has not been collected. Report sex- and gender-based analyses where performed, justify reasons for lack of sex- and gender-based analysis.*
Reporting on race, ethnicity, or other socially relevant groupings	*Please specify the socially constructed or socially relevant categorization variable(s) used in your manuscript and explain why they were used. Please note that such variables should not be used as proxies for other socially constructed/relevant variables (for example, race or ethnicity should not be used as a proxy for socioeconomic status). Provide clear definitions of the relevant terms used, how they were provided (by the participants/respondents, the researchers, or third parties), and the method(s) used to classify people into the different categories (e.g. self-report, census or administrative data, social media data, etc.) Please provide details about how you controlled for confounding variables in your analyses.*
Population characteristics	*Describe the covariate-relevant population characteristics of the human research participants (e.g. age, genotypic information, past and current diagnosis and treatment categories). If you filled out the behavioural & social sciences study design questions and have nothing to add here, write "See above."*
Recruitment	*Describe how participants were recruited. Outline any potential self-selection bias or other biases that may be present and how these are likely to impact results.*
Ethics oversight	*Identify the organization(s) that approved the study protocol.*

Note that full information on the approval of the study protocol must also be provided in the manuscript.

Field-specific reporting

Please select the one below that is the best fit for your research. If you are not sure, read the appropriate sections before making your selection.

☐ Life sciences ☐ Behavioural & social sciences ☒ Ecological, evolutionary & environmental sciences

For a reference copy of the document with all sections, see nature.com/documents/nr-reporting-summary-flat.pdf

Ecological, evolutionary & environmental sciences study design

All studies must disclose on these points even when the disclosure is negative.

Study description	An analysis of eukaryote DNA recovered from metagenomes sequenced from sediments collected from Holocene Adélie penguin (Pygoscelis adeliae) colonies dating back 6,000 years along the Ross Sea coast, Antarctica
Research sample	We excavated 14 pits at ten Adélie penguin colony sites along the western margin of the Ross Sea in January 2019 and January-February 2020. These included Cape Bird, Cape Royds (2 pits), Cape Barne (2 pits), Cape Crozier and Marble Point (2 pits) in the southern Ross Sea, Campo Icaro, Adélie Cove and North of Adélie Cove in the mid-Ross Sea, and Cape Hallett and Cape Adare (2 pits) in the northern Ross Sea. Of these, the colonies at Cape Bird, Cape Royds, Cape Crozier, Adélie Cove, Cape Hallett and Cape Adare are currently active, while Cape Barne, Marble Point, Campo Icaro and North of Adélie Cove are sites of former but now abandoned colonies.
Sampling strategy	Excavations at active colonies were located on abandoned nest mounds outside the current extent of the colony. Placement of excavations at abandoned colonies was determined by digging small test holes on suspected abandoned nest mounds to confirm the presence of buried ornithogenic soil horizons. Excavated pits measured 0.09-1.0m2 in area. Most excavations were continued until permafrost ice was encountered, preventing further excavation with hand tools. Excavations were performed using trowels and sediment was removed in spits of 5cm depth when no clear stratigraphic boundaries were present. Bones, eggshell and desiccated chick remains were recovered by sieving sediment in the field and are stored with the bulk sediment samples. Field sterility protocols that are critical for ancient DNA research were adhered to, including thorough cleaning of tools, tarpaulins and other equipment between sites using DNA AWAY™ Surface Decontaminant (Thermofisher). Sediment samples for DNA analysis were collected from freshly exposed faces at the side of pits, by pushing a sterile 50mL Falcon tube directly into the sediment or by scooping with a stainless steel spatula (cleaned between samples using DNA AWAY) and tubes remained sealed until they were subsampled within an ancient DNA laboratory.
Data collection	Field data (e.g. stratigraphic notes) were taken at the time of excavation in field notebooks by JRW. GPS coordinates were measured

Data collection	by JRW using a hand-held Garmin GPSMap device. DNA sequencing was undertaken at BGI_Shenzen using the MGISEQ-2000 platform.
Timing and spatial scale	Sediment samples were collected over two summer field-seasons (January 2019 and January-February 2020). Radiocarbon dating indicates sample ages extend from modern back to around 6,000 years. Our study sites extended over ~800 km, from Cape Barne in the southern Ross Sea to Cape Adare in the northern Ross Sea.
Data exclusions	No data were excluded from the analysis
Reproducibility	In several of the analyses mitochondrial and mitochondrial + nuclear DNA were assessed seperately, and two taxonomic assignment methods were used, to verify results.
Randomization	Not relevant
Blinding	Not relevant

Did the study involve field work? ☒ Yes ☐ No

Field work, collection and transport

Field conditions	Day trips from Scott Base and Zucchelli Station via helicopter or vehicle; walking from Cape Bird Hut; Field camp at Cape Hallett. Temperatures typically 0 - minus 7 degrees C. Most excavations undertaken in calm wind conditions.
Location	Coast of East Victoria Land, Antarctica, between Cape Adare (71.30219444 S) in the north and Cape Barne (77.5777833 S) in the south, and Marble Point to the east (163.833717 E) and Cape Hallett to the west (170.22511111 E).
Access & import/export	All research planning was undertaken in conjunction with Antarctica New Zealand and was subject to an Environment Evaluation for activities pursuant to section 17 of the Antarctica (Environmental) Protection Act 1994. Sample collection and entry to Antarctic Specially Protected Areas - ASPAs (121, 124, 155, 157) for excavation or transiting were undertaken with the appropriate permissions and permits. Samples were imported to New Zealand under a "Permit to import laboratory specimens" (Permit No. 2018067364) granted to Landcare Research Ltd and are stored frozen at the Manaaki Whenua Landcare Research Soil Ecology & LTEL Transitional Facility, Lincoln.
Disturbance	All grey water, refuse and human waste was collected and transported to Scott Base or Zucchelli Station for appropriate treatment and disposal. All external field clothing was issued by Antarctica New Zealand avoiding contamination from New Zealand field sites. Socks and under garments were thoroughly cleaned before field deployment. Team members followed New Zealand Department of Conservation quarantine procedures and made checks of these items before departure to Antarctica. All food was provided by Antarctica New Zealand. Prior to deployment all boots and equipment were thoroughly cleaned. All excavation equipment and field gear was thoroughly washed prior to travel to Antarctica. Soil from all excavation equipment was removed and gear was thoroughly cleaned in between sites. Excavated material was sieved onto tarpaulins and any soil not collected was returned to the pit after excavation were complete. Surface stones were kept separately and returned to the surface after the excavation to ensure that the site appears as it did prior to excavation. Areas with obvious biological activity (e.g. mosses) on surface were avoided.

Reporting for specific materials, systems and methods

We require information from authors about some types of materials, experimental systems and methods used in many studies. Here, indicate whether each material, system or method listed is relevant to your study. If you are not sure if a list item applies to your research, read the appropriate section before selecting a response.

Materials & experimental systems

n/a	Involved in the study	
☒	☐	Antibodies
☒	☐	Eukaryotic cell lines
☐	☒	Palaeontology and archaeology
☒	☐	Animals and other organisms
☒	☐	Clinical data
☒	☐	Dual use research of concern
☒	☐	Plants

Methods

n/a	Involved in the study	
☒	☐	ChIP-seq
☒	☐	Flow cytometry
☒	☐	MRI-based neuroimaging

Palaeontology and Archaeology

Specimen provenance	Sample collection was undertaken with the appropriate permissions and permits. Samples were imported to New Zealand under a "Permit to import laboratory specimens" (Permit No. 2018067364) granted to Landcare Research Ltd and are stored frozen at the

	Manaaki Whenua Landcare Research Soil Ecology & LTEL Transitional Facility, Lincoln.
Specimen deposition	Sediment samples and DNA extracts are held by the Long-Term Ecology Laboratory, Manaaki Whenua Landcare Research, Lincoln, New Zealand.
Dating methods	Fragments of air-dried penguin eggshell (weighing 17.6 – 52.8 mg in total) were selected from individual sediment spits and radiocarbon dated at the Keck Carbon Cycle AMS facility at the University of California, Irvine, by accelerator mass spectrometry (AMS). Samples were leached 50% with dilute HCl prior to hydrolysis with 85% phosphoric acid. Sample preparation backgrounds were subtracted, based on measurements of 14C-free calcite. Results were corrected for isotopic fractionation according to the conventions of Stuiver & Polach, with d13C values measured on prepared graphite using the AMS spectrometer. Small CO2 yields from hydrolysis resulted in larger than expected uncertainties for samples UCIAMS#229695, 229708, 229712, 229714, 229715 and 229717.

☒ Tick this box to confirm that the raw and calibrated dates are available in the paper or in Supplementary Information.

Ethics oversight	No ethical approval was required

Note that full information on the approval of the study protocol must also be provided in the manuscript.

Plants

Seed stocks	*Report on the source of all seed stocks or other plant material used. If applicable, state the seed stock centre and catalogue number. If plant specimens were collected from the field, describe the collection location, date and sampling procedures.*
Novel plant genotypes	*Describe the methods by which all novel plant genotypes were produced. This includes those generated by transgenic approaches, gene editing, chemical/radiation-based mutagenesis and hybridization. For transgenic lines, describe the transformation method, the number of independent lines analyzed and the generation upon which experiments were performed. For gene-edited lines, describe the editor used, the endogenous sequence targeted for editing, the targeting guide RNA sequence (if applicable) and how the editor was applied.*
Authentication	*Describe any authentication procedures for each seed stock used or novel genotype generated. Describe any experiments used to assess the effect of a mutation and, where applicable, how potential secondary effects (e.g. second site T-DNA insertions, mosiacism, off-target gene editing) were examined.*
